# Advanced *In vivo* Use of CRISPR/Cas9 and Anti-sense DNA Inhibition for Gene Manipulation in the Brain

**DOI:** 10.3389/fgene.2015.00362

**Published:** 2016-01-12

**Authors:** Brandon J. Walters, Amber B. Azam, Colleen J. Gillon, Sheena A. Josselyn, Iva B. Zovkic

**Affiliations:** ^1^Department of Neuroscience and Mental Health, The Hospital for Sick ChildrenToronto, ON, Canada; ^2^Department of Psychology, University of Toronto MississaugaMississauga, ON, Canada; ^3^Department of Physiology, University of TorontoToronto, ON, Canada

**Keywords:** CRISPR/Cas9, anti-sense nucleotides, Morpholino, gene editing, brain, CNS, knockdown, overexpression

## Abstract

Gene editing tools are essential for uncovering how genes mediate normal brain–behavior relationships and contribute to neurodegenerative and neuropsychiatric disorders. Recent progress in gene editing technology now allows neuroscientists unprecedented access to edit the genome efficiently. Although many important tools have been developed, here we focus on approaches that allow for rapid gene editing in the adult nervous system, particularly CRISPR/Cas9 and anti-sense nucleotide-based techniques. CRISPR/Cas9 is a flexible gene editing tool, allowing the genome to be manipulated in diverse ways. For instance, CRISPR/Cas9 has been successfully used to knockout genes, knock-in mutations, overexpress or inhibit gene activity, and provide scaffolding for recruiting specific epigenetic regulators to individual genes and gene regions. Moreover, the CRISPR/Cas9 system may be modified to target multiple genes at one time, affording simultaneous inhibition and overexpression of distinct genetic targets. Although many of the more advanced applications of CRISPR/Cas9 have not been applied to the nervous system, the toolbox is widely accessible, such that it is poised to help advance neuroscience. Anti-sense nucleotide-based technologies can be used to rapidly knockdown genes in the brain. The main advantage of anti-sense based tools is their simplicity, allowing for rapid gene delivery with minimal technical expertise. Here, we describe the main applications and functions of each of these systems with an emphasis on their many potential applications in neuroscience laboratories.

Genes exert powerful effects on human behavior, and maladaptive alterations in gene function are implicated in a diverse range of neurological and neuropsychiatric disorders. Uncovering the mechanisms by which genes influence behavior is an important goal of neuroscience. In addition to elucidating the roles genes play in normal regulation of cognitive and affective functions, this understanding is key for developing novel and effective therapeutic interventions for brain disorders (Mamdani et al., 2013; Bae et al., 2015; Szyf, 2015). Achieving this goal requires the ability to manipulate (both increase and decrease) gene expression. The regulation of gene function in the brain is highly dynamic and may be different across brain regions (Zovkic et al., 2013; Walters and Zovkic, 2015). Therefore, manipulating gene function in the brain poses particular challenges, in that gene manipulations should be temporally and spatially specific.

Many gene editing tools have been developed to address these challenges. Here, we will discuss some current gene editing tools that hold particular promise for application in neuroscience. In particular, we will focus on the CRISPR/Cas9 system, which allows for a wide range of gene editing applications through an unprecedented degree of flexibility and user-friendly engineering technology. In addition, we will discuss the latest advances in anti-sense oligonucleotide approaches for gene knockdown because of their high level of accessibility and ease with which they can be applied across neuroscience laboratories with minimal requirements for in-house design.

## The evolution of gene editing tools

Perhaps the earliest efforts at manipulating gene function to understand their role in behavior were pharmacological. Drugs have been used as protein agonists or antagonists to provide a temporally restricted (and regionally restricted, if microinjected into discrete brain regions) method of manipulating the function of a protein. While drugs offer good temporal precision, they also have some drawbacks, such as off-target effects. In addition, it is difficult to design new drugs to target new proteins of interest. The development of knockout transgenic mice, which provided the ability to target any gene of interest (Hanahan et al., 2007), has allowed researchers to specifically manipulate a variety of genes. The first knockout transgenic strategy used germline knockout mutations that resulted in transgenic mice in which the target gene was either deleted or overexpressed in all tissues, typically throughout development. The use of tissue-specific promoters, such as the brain-specific t9 (Bessis et al., 1997) or α CaMKII promoter (Mayford et al., 1996), to drive transgene expression in particular tissue types afforded some regional specificity of overexpression. Nevertheless, the temporal specificity was still lacking, as transgene expression was tied to the transcriptional onset of the promoter driving its expression. Moreover, these transgenic approaches required both considerable time and effort to generate and breed.

The next generation of transgenic strategies involved tissue-specific gene knockout. This was achieved through the use of Cre-LoxP system (Gu et al., 1994). Cre recombinase is an enzyme that splices out genes flanked by LoxP sites only in the regions where Cre is expressed, thereby achieving tissue-specific gene deletion when Cre-expressing mice are crossed with mice containing LoxP sites around the gene of interest (see (Kos, 2004) for full review of Cre-LoxP mice). The ability to regulate Cre expression with diverse tissue-specific promoters provided better tissue selectivity of the knockout, but temporal control was still tied to the promoter driving Cre expression.

Later modifications to Cre-LoxP system had led to an inducible form of Cre that substantially improved temporal specificity in transgenic mice. Specifically, the activity of Cre was engineered to depend on either the introduction of a synthetic hormone or the doxycycline system, whereby Cre activity and the resulting genetic knockout occur only after the activating compound is delivered (Guo et al., 2005; Feil et al., 2009). Inducible Cre systems provide experimental control over the onset of gene deletion (to within days or weeks following delivery of the activating compound), and at the same time ensuring normal gene function during development (Michel et al., 1979; Guo et al., 2005; Feil et al., 2009; Wilson et al., 2014). In addition to these types of transgenic strategies, wherein Cre is provided by a transgenic mouse, various viral methods have been developed to exogenously provide Cre activity by microinjection of a viral vector. When administered to transgenic mice with LoxP sites flanking the gene of interest, this method further extended the temporal and spatial specificities of the knockout, although it requires the additional step of viral production and stereotaxic surgery for targeted viral delivery (Sinnayah et al., 2004). One disadvantage of these systems, however, is the necessity to develop and maintain transgenic mouse lines expressing floxed genes of interest and the difficulty in targeting multiple genes simultaneously.

In parallel with advancements in transgenic mice, various RNA interference (RNAi) techniques were developed to decrease gene expression in wild-type rodents without requiring maintenance of large transgenic mouse colonies. RNAi makes use of an endogenous pathway within most eukaryotic cells, whereby double-stranded RNA is processed by the enzyme Dicer and loaded into a RISC (RNA-induced silencing complex), which recognizes RNA transcripts that share homology with the RNA loaded into the RISC (Wilson and Doudna, 2013). Although the experimentally determined RNA introduced to this system can take many forms, shRNA is most commonly utilized in neuroscience. In this system, shRNA designed to target a specific transcript is overexpressed in particular brain regions through the use of viral vectors, mostly commonly adeno-associated viral vectors. An advantage of this approach is that it can be performed in wild-type rodents and the degree of gene knockdown can be experimentally controlled. In addition to target-specific brain regions, specific cell types may also be targeted through the use of different AAV serotypes (Choi et al., 2005) or different types of viruses, such as herpes simplex virus (HSV; Burton et al., 2002). Although designing the shRNA may be less time and labor intensive than creating new lines of transgenic mice, the procedure is not trivial. Typically, many different shRNAs are individually designed and validated before sufficient knockdown is obtained (Choi et al., 2005). A primary disadvantage of this approach is the potential for off-target effects, which hampers the interpretation of results obtained from shRNA studies (Birmingham et al., 2006; Jackson and Linsley, 2010; Moore et al., 2010).

Each new tool we discussed exhibits improvement over previously available methods, but extensive design and time requirements of each strategy limit flexible switching to new gene targets. Here, we discuss the next generation of genetic tools, focusing first on recent advancements in the highly flexible CRISPR/Cas9 system, which enables simple “plug and play” mechanics to study many different genes simultaneously. Second, we focus on removing the need for viruses/mouse models by highlighting the advances made in anti-sense nucleotides, mainly anti-sense oligonucleotides (ASOs) and Morpholinos, which can be directly added to the brain without any delivery system, to effect gene regulation.

## Using CRISPR/Cas9 for gene modulation in the brain

The discovery of the CRISPR/Cas9 system and its adaption for use in gene editing has sparked a revolution in many scientific fields due to its ability to edit specific sequences in the genome. Initially, this may seem no more advantageous then various RNAi methods already in use. However, the true power of the CRISPR/Cas9 system lies in is its simplicity and flexibility. Whereas RNAi development can be time consuming and/or costly, often requiring many iterations before producing an acceptable level of knockdown (Moore et al., 2010), nearly every targeting sequence designed for use with CRISPR/Cas9 has a high chance of producing the desired knockout (Cong et al., 2013; Mali et al., 2013). This efficiency allows for rapid and effective switching between gene targets and importantly, for developing multiple targeting sequences to edit multiple genes simultaneously. In addition, CRISPR/Cas9 tools can be easily developed in individual laboratories with minimal need for specialized knowledge. Finally, the CRISPR/Cas9 system is very user-friendly, with fully-accessible resources for generating targeting constructs available at no cost (See http://www.genome-engineering.org for in-depth CRISPR/Cas9 protocols and resources).

CRISPR/Cas9 is a system in which an RNA-guided riboprotein, Cas9, cleaves DNA at genomic sites that are specified by a target sequence encoded within CRISPR. The system was discovered as a bacterial/archaea immune response, which defends the organism against viral infections by incorporating short segments of invading DNA within specialized regions of the genome called CRISPR (clustered regularly interspaced short palindromic repeats). The short fragments of foreign DNA contained within CRISPR act as a guide that targets invading DNA through association with Cas9 (CRISPR-associated protein 9), a nuclease that uses the guide sequence to detect and cut any DNA that matches the foreign fragment encoded in the CRISPR array. By co-opting and engineering this system, scientists are able to dictate which fragments are “foreign,” or rather which genes/fragments will be targeted for experimental purposes (Cong et al., 2013; Mali et al., 2013). This system allows for the introduction of double-stranded DNA breaks (dsDNA) at nearly any specified sequence within the genome (for an in-depth look at the Cas9 system, see review, Hsu et al., 2014). Once introduced, double stranded DNA breaks can be utilized to create gene knockouts or knock-ins at the location of the double-stranded DNA break, as described below.

### Using CRISPR/Cas9 for gene knockout

The process of knocking out a gene using CRISPR/Cas9 is both straight-forward and fast. First, an analog of the CRISPR guide region found in bacterial systems must be created to identify the gene region that is to be targeted. This is achieved by creating a single guide RNA (sgRNA), which is used to direct Cas9 to the gene of interest, where it causes a dsDNA break. In order for this to occur, the region to be cut must also contain a PAM (protospacer adjacent motif), a simple nucleotide sequence consisting of any base followed by two Gs. Once a cut-site is created, it initiates dsDNA break repair by the non-homologous end joining (NHEJ) pathway in the brain, which frequently results in insertion/deletion mutations (INDELs; Heidenreich et al., 2003). In cases where INDELS do not form immediately, Cas9/sgRNA will rebind that location and cut again, increasing the likelihood of successful INDEL formation. Introducing INDELS particularly to the coding regions of target genes causes frameshift mutations that form the basis of CRISPR/Cas9-based gene knockout (Streisinger et al., 1966). This system has several advantages over previous approaches, including the overall ease of use, with no specialized techniques required to create and validate the setup by end-user labs. For example, a laboratory needs only to know basic molecular cloning and cell culture techniques to create and validate the sgRNA.

In addition to the relative ease of developing CRISPR/Cas9 to achieve highly efficient gene editing, this system can be easily adapted to target multiple genes simply by creating additional sgRNA sequences directed against different gene targets (Shalem et al., 2014). This may be important for studying the effects of several gene products or pathways in behavior, or for deleting proteins that are encoded by multiple genes. Similarly, gene targets can be switched if additional regulatory genes are identified, with minimal effort. The use of Cas9 to induce gene deletions in the brain is less common than its use in other cell types. However, studies have shown that CRISPR/Cas9 can be used to delete genes in the brain (for example, *Mecp2*; Swiech et al., 2015). In this example, knockout was achieved through the use of two AAVs, one to express Cas9 and one to express the sgRNA and GFP. These viruses were mixed and injected into the hippocampus where they resulted in ~80% co-transduction rate and up to a 70% reduction in Mecp2-positive cells in the dentate gyrus. When memory was assayed using contextual fear conditioning, Mecp2-mutated mice showed reduced freezing behavior, indicating poor memory and thus illustrating the utility of the Crispr/Cas9 approach in mice behavioral studies.

Swiech et al. (2015) further demonstrated the power of CRISPR/Cas9 for exploring functional outcomes of multiple genes with overlapping functions. Specifically, the authors developed three sgRNAs targeted against key DNA methyltransferase enzymes, Dnmt1, Dnmt3a, and Dnmt3b. They targeted each with a sgRNA and showed simultaneous co-knockouts of each one (% reduction in Dmnt+ cells: 60, 42, and 17% for Dnmt1, 3a, and 3b, respectively). This study provided the first evidence of simultaneous multi-gene knockout in the brain with only the addition of multiple sgRNAs to the system, demonstrating the feasibility of this approach in neuroscience. Consistent with previous studies using classical knockout approaches (Feng et al., 2010), Swiech et al. found no behavioral effects when each gene was knocked down alone. However, when multiple genes were knocked out simultaneously using the multiplexed CRISPR/Cas9, mice showed poor memory.

Observing a memory phenotype only with simultaneous knockdown of multiple DNMTs demonstrates the functional significance of this system and the potential for multi-gene targeting with CRISPR/Cas9 in the brain. However, this study also reveals a marked decrease in knockdown efficiency with an increased number of sgRNAs. Specifically, the knockdown efficiency was reduced from 70% when only one gene was targeted (MecP2) to 60, 42, and 17% when three DNMTs were targeted simultaneously. It is unclear whether this loss in efficiency is indicative of typical outcomes of multiple gene deletions in the brain, or if this effect is locus specific, as local chromatin configuration may influence the efficiency of the sgRNA–Cas9 complex (Wu et al., 2014a). *In vitro*, thousands of sgRNA–Cas9 complexes have been multiplexed to enable high-throughput screening (Shalem et al., 2014, 2015; Zhou et al., 2014), demonstrating the capacity of the system to handle many targets. Furthermore, multi-target studies in the brain are needed to better characterize the efficacy of multi-gene knockdown, but the potential of CRISPR/Cas9 to knockout genes of interest in neuroscience remains vast.

### Use of Cas9 for knock-in mutations

As briefly mentioned above, CRISPR/Cas9 is a flexible and versatile system with the potential for multiple gene editing functions, including the introduction of knock-in mutations. Knock-in mutations may be of particular interest for understanding disease states in which particular mutations have been identified. As with gene knockout, dsDNA breaks caused by Cas9 can also be harnessed to produce knock-in mutations, typically through the homology-dependent repair (HDR) pathway (Ran et al., 2013a). This pathway is a normal DNA repair pathway that repairs dsDNA breaks by comparing the region containing a dsDNA break with a nearby homologous region, typically a sister chromatid, and repairs the dsDNA break using the homologous region as a template. This process can be co-opted to achieve high knock-in levels in regions targeted by sgRNA by introducing an engineered DNA sequence flanked by homologous regions that serve as repair templates (Ran et al., 2013a).

The main limitation of this mechanism for neuroscience applications is its reliance on HDR, which is not usually present in terminally differentiated cell types like neurons (Ran et al., 2013a). For this reason, efforts to produce targeted knock-ins using HDR-based tools in neurons have yet to be successful. However, HDR-based knock-ins have successfully introduced gene mutations *in vivo* in the lungs, resulting in nearly equal frequency of knock-in mutations when compared to INDEL-based knockouts (Platt et al., 2014). Nonetheless, if efforts to transition HDR-based mutations to neurons fail, efforts to harness the NHEJ pathway, which is found in the brain, show some promise for producing knock-in mutations (Maresca et al., 2013; Auer et al., 2014), although this approach has not yet been demonstrated in neurons. Interestingly, Cpf1, an enzyme similar to Cas9, is a newly characterized member of the Cas family. Similar to Cas9, Cpf1 causes double-stranded DNA breaks, but unlike Cas9, the DNA break results in overhanging “sticky ends” that promote NHEJ-based knock-ins (Maresca et al., 2013; Zetsche et al., 2015). These advancements suggest that Cpf1 may be a solution for obtaining efficient knock-in mutations in the nervous system (Platt et al., 2014). This approach has many potential applications that would allow various forms of mutations, including disease-specific mutations found in humans, as well as loxP sites for gene deletion, to be introduced directly into the nervous system. The feasibility and utility of such applications will depend on their validation at sufficiently high efficiency to make them useful for *in vivo* work.

While CRISPR/Cas9 has most commonly been used for direct gene editing, this system may also be used to modulate gene expression without editing the genome directly. Two primary methods have been developed for indirect regulation of gene activity, each relying on a mutated form of Cas9 that lacks nuclease activity (dCas9; Cheng et al., 2013; Gilbert et al., 2013; Maeder et al., 2013). The two methods vary in the components modified, with one modifying the dCas9 and the other modifying the sgRNA (Cheng et al., 2013; Gilbert et al., 2013; Maeder et al., 2013; Konermann et al., 2015). Irrespective of the target, both modifications operate on the same basic premise: instead of using sgRNA–Cas9 to cut DNA, the sgRNA–Cas9 is used as a scaffold for other modifying enzymes to be recruited to the targeted locus to modify its function.

### Using sgRNA/Cas9 as a scaffold to inhibit or activate genes

sgRNAs can target almost any site within the genome with excellent selectivity, suggesting that sgRNA–dCas9 complexes can also be targeted to specific regulatory positions of a given gene. Indeed, recent studies demonstrated either promoter- or enhancer-selective targeting of sgRNA–dCas9, which was used as a scaffold for recruiting transcriptional activators or repressors to the designated target region, thereby modifying the gene's transcriptional activity (Shalem et al., 2015). This scaffolding function can be achieved with multiple approaches either by fusing the transcriptional modulator directly to dCas9 (Cheng et al., 2013; Gilbert et al., 2013; Maeder et al., 2013; Perez-Pinera et al., 2013) or by fusing a repeated motif to dCas9 to attract multiple copies of the endogenous modulator to a locus (Tanenbaum et al., 2014). Here, we will focus our attentions on a third option, in which the sgRNA itself is modified to act as a scaffold. This latter option represents the most flexible and robust method of recruiting particular factors to the gene of interest with CRISPR/Cas9.

**Figure 1:**
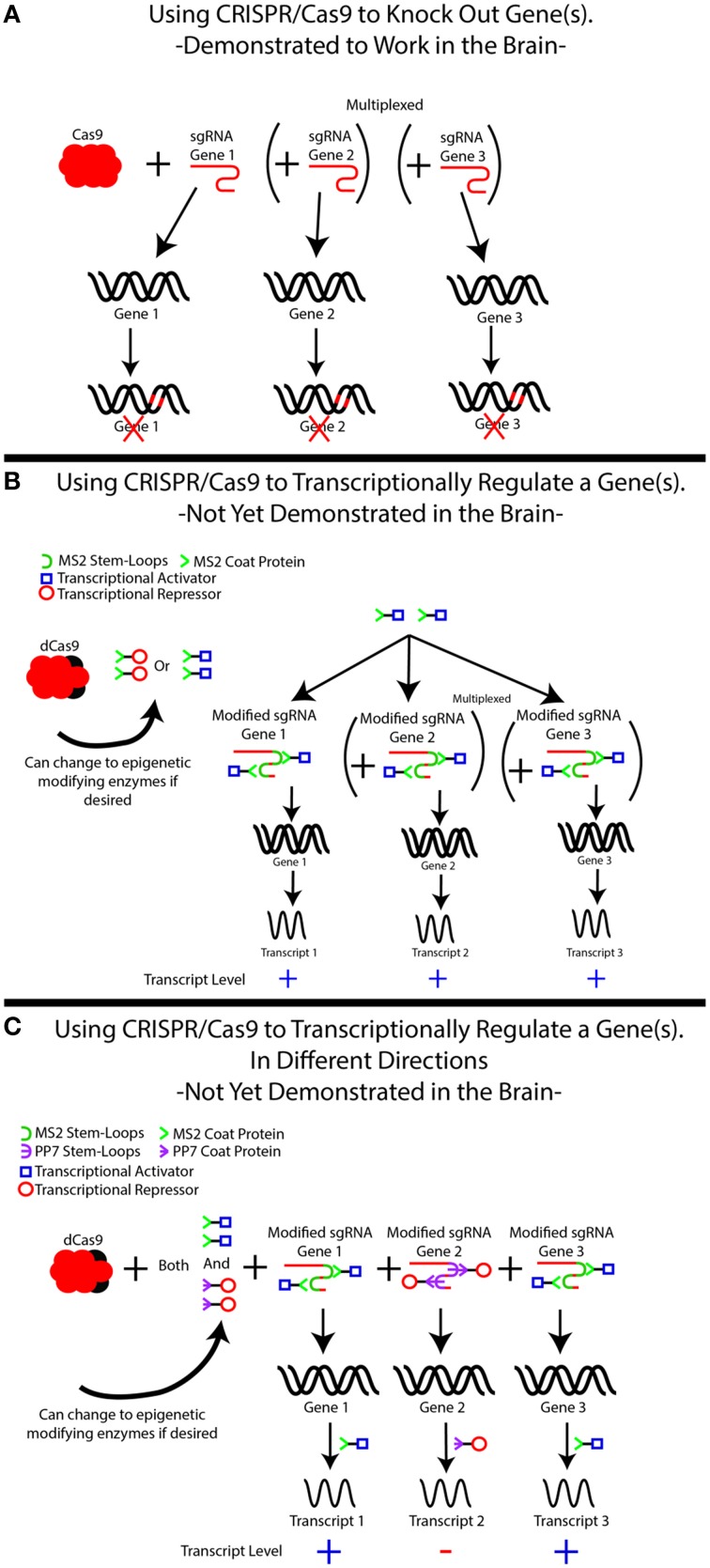
**The use of CRISPR/Cas9 for simplexed and multiplexed gene modulation**. **(A)** CRISPR/Cas9-based gene knockouts have been demonstrated in the brain in both simplexed (1 target) and multiplexed (≥2 genes) fashions. Although our example demonstrates three gene targets, additional targets may be added. In each case, knockdown is achieved by creating a single guide RNA (sgRNA) specific to the gene of interest, which recruits Cas9 to cut the DNA in order to induce frameshift mutations that knock out the gene. Frameshift mutations are illustrated as red marks on the DNA. The use of multiple sgRNAs can induce knockouts of multiple genes simultaneously. **(B)** An inactive form of Cas9, known as dCas9, can be used to modulate gene expression by recruiting transcriptional regulators to genes of interest, but this system has yet to be demonstrated in the brain. In this system, the sgRNA–dCas9 molecule is treated as a scaffold for recruiting other proteins, most commonly transcriptional activators (blue squares) or transcriptional repressors (red circles) to the specified location within the genome. This is not limited to transcriptional regulators and can easily be modified to bring epigenetic-modifying enzymes or specific transcription factors to these locations. This is accomplished by modifying the sgRNA to include MS2 sequences (green half circle), which is recognized by the MS2 coat protein, MCP (green >). The MS2 coat protein is fused to the protein of interest, in this case either a transcriptional activator or repressor. The MS2-fusion protein is transduced into the cells of interest in addition to the dCas9 and the modified sgRNA, directing the activator/repressor to the specific loci. It is important to note that with only an MS2-fusion construct, only 1 type of fused protein can be delivered. That is, either transcriptional activation or repression can occur, but not both. Simultaneous multiplexing of repressors and activators to distinct loci is demonstrated in **(C)**, where additional stem loops (the PP7 stem loop, purple **϶**) and stem loop binding proteins (PP7 binding protein, purple 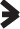) are added to the system and fused to specific DNA-modifying enzymes. This allows separate proteins to be recruited to distinct loci, achieving mixed activation/repression of different genes.

Many types of proteins have evolved to bind specific RNA sequences, including MS2 coat protein (MCP). MCP binds to RNA through an MS2 stem loop formed by a specific RNA sequence. Such stem loop structures can be engineered into endogenous loops in tracrRNA, a component of sgRNA that recruits Cas9. These stem loops are recognized by viral coat proteins, such as MCP, which can be engineered to fuse with transcriptional activators or repressors. Fusing the transcriptional activator HSF1 to MCP has been used to achieve robust (>100x) activation of target genes (Figure 1). Similarly, pairing this complex with transcriptional repressors results in robust inhibition (>80%; Gilbert et al., 2014; Konermann et al., 2015), demonstrating a high degree of efficacy for modifying gene function.

One advantage of engineered sgRNAs is the potential to multiplex distinct and multiple gene loci to repressors or activators simultaneously, such that some genes are turned off in the same model in which other genes are turned on. By relying on different stem loops that are recognized by different proteins [PP7 stem loop, PP7 coat protein; PCP Lim and Peabody, 2002; Shalem et al., 2015; Zalatan et al., 2015], one can ensure that certain sgRNAs activate their loci by pairing them with MS2 loops, while engineering other sgRNAs to repress their loci by pairing them with PP7 loops (Zalatan et al., 2015). Activation and overexpression of distinct gene products are achieved by simultaneously expressing MCP fused to a transcriptional activator and PCP fused to a transcriptional repressor. A major advantage of this approach is the ability to induce opposing regulatory effects on different genes, a strategy that can be easily scaled up to include any number of loci because of the small size of sgRNAs (Zalatan et al., 2015). The ability to active and silence different genes is important in investigating the neural basis of behavior.

One novel application of the scaffolding approach is the ability to create position-specific epigenetic modifications at behaviorally relevant genes. Broader accessibility of genome-wide sequencing approaches has redefined our understanding of epigenetic modifications, highlighting the crucial role of gene- and region-specific effects of epigenetic modifications on gene activity (Bargaje et al., 2012; Shlyueva et al., 2014; Zovkic et al., 2014). Until recently, the study of epigenetics relied almost exclusively on pharmacological tools that resulted in global effects across all genes in the brain region of interest. However, recent work using dCas9 as a scaffold showed a high capacity to regulate epigenetic changes at particular genes, as well as particular regulatory regions of the gene. Specifically, in the first study demonstrating the feasibility of this approach, the core domain of the p300 histone acetyltransferase enzyme was fused to dCas9 and targeted to either promoter or enhancer regions of genes by using region-specific sgRNAs (Hilton et al., 2015). This resulted in site-specific changes in histone acetylation and transcription (Hilton et al., 2015), providing the first direct evidence that selective changes in histone acetylation at gene promoters support gene activity. This innovation expands the toolbox available for studying the complex interaction between genes, epigenomics, and behavior.

While Cas9-based gene editing has been successfully demonstrated *in vivo* in the nervous system (Platt et al., 2014; Swiech et al., 2015), the use of CRISPRi (using dCas9 to repress loci) or CRISPRa (using dCas9 to activate loci) has not been demonstrated in the brain. However, there is no reason that these methods will not translate into the CNS, since the main requirement for the system to work is for Cas9/sgRNA to bind to a gene locus, which has already been demonstrated to occur in the brain with wild-type Cas9 (Platt et al., 2014; Swiech et al., 2015). In addition, these methods can even be combined with light-activation domains, giving greater temporal specificity to the transcriptional manipulations (Wu et al., 2010; Konermann et al., 2013; Nihongaki et al., 2015; Polstein and Gersbach, 2015). The major limitation to these methods is the need for additional constructs, such as the MCP-binding protein fused to the desired enzyme and either additional AAVs or viruses with large packing limits, such as herpes simplex virus (HSV). The use of viruses to deliver CRISPR/Cas9 in the brain is discussed further in the sections given below.

## Off-target effects of CRISPR/Cas9

Although CRISPR/Cas9 primarily targets specific loci within the genome, some off-target activities have been reported at sites homologous to the sgRNA sequence (see Wu et al., 2014b for an in-depth review). The frequency of off-target binding depends on the guide sequence, with some sgRNAs having as few as 10 off-target sites in the genome, and others having many more (Kuscu et al., 2014; Frock et al., 2015). If off-target effects are of particular concern, then they can be handled in a number of ways. First, the guides themselves can be optimized to reduce the number of homologous targets in the genome. Off-target effects can occur because the Cas9–sgRNA complex can tolerate between 3 and 5 mismatches in the guide, while still retaining some functional capacity at off-target sites (Fu et al., 2014). Therefore, designing sgRNAs that minimize homology to other sites is the simplest strategy for reducing off-target effects. Other approaches include decreasing the size of the sgRNA from the traditional 20 to 17–19 bp, which can reduce or eliminate off-target activity without negatively affecting the activity at the target site (Fu et al., 2014). Modifications can also be done to Cas9, as evidenced by eCas9, a mutated form of Cas9 that exhibits vastly decreased off-target activity compared to wild-type Cas9 (Slaymaker et al., 2016). Perhaps the best way to overcome potential off-target effects is to simply verify any observed results with two separate sgRNAs for the same gene, ensuring that the genes do not have homologous sgRNAs. Alternatively, appropriate targeting can be confirmed by rescuing the deletion phenotype by re-introducing the deleted gene with overexpression approaches.

Other strategies for reducing off-target effects include using a system in which two sgRNAs must bind the same region to achieve full nuclease activity, using either paired nickases (Ran et al., 2013b) or FokI (Guilinger et al., 2014; Tsai et al., 2014; Wyvekens et al., 2015). The paired nickase approach is based on the observation that two separate sites within Cas9 cut each strand of DNA to achieve the DSBs needed for gene deletion. Mutating one of these sites to prevent cleavage ensures that one sgRNA/“nickase” pair can nick the DNA only on a single strand, thus precluding INDEL formation. However, when two nickases directed against opposite strands of DNA occur in close proximity, DSBs can be induced and gene deletion can occur. This added requirement for two sgRNAs within the same target region effectively reduces off-target activity. The Fok1 method follows the same principle, but instead of using a nickase, Cas9 is mutated to prevent any nuclease activity (dCas9). The dCas9 is then fused to a Fok1 monomer to produce fCas9. Fok1 has no nuclease activity as a monomer, but it readily cleaves DNA upon dimerization. Therefore, by designing two sgRNAs approximately 20bp apart, both fCas9s will bind within the same region, allowing Fok1 to dimerize and induce a DSB. Although these approaches greatly reduce off-target activity, they require the design of additional vectors and may require more viruses to deliver modified Cas9s and additional sgRNA, making it cumbersome for *in vivo* use in the brain.

## Delivering CRISPR–Cas9 into the brain

Although there are many advantages of CRISPR/Cas9 as a gene editing tool in the brain, the application of this technology in neuroscience has been slow. One reason for this may be the difficulty of delivering Cas9 into the brain. Cas9 is a large protein, with the promoter and coding sequence spanning more than 4 kb in size, which itself can fill the packaging limit of AAVs. When combined with sgRNAs and the fluorophores required to track its expression, the experiment can easily exceed the packaging limit of AAV vectors, which typically ranges from 4 to 5 kb (Wu et al., 2010). Nevertheless, multiple strategies have been developed to overcome this barrier, and we will discuss three of the most viable solutions here.

One simple solution is offered by the development of a Cas9 transgenic mouse that has Cas9 (or a Cre-mediated Cas9) knocked into the ROSA26 locus (Platt et al., 2014), which is commonly used to drive robust and tissue-wide gene expression (Casola, 2010). The Cas9 mouse eliminates the issue of Cas9 packaging and allows viral vectors to be used exclusively to produce sgRNA or multiple sgRNAs, as well as any other experimentally required proteins. This method has been successfully utilized to knockout genes in the brain and even to drive knock-in mutations in the lungs (Platt et al., 2014). Unfortunately, this method precludes the use of tool kits based on dCas9, including CRISPRi or CRISPRa, which would require the creation of an additional dCas9 transgenic line. Another limitation of this solution is the need to maintain a dedicated mouse line, which eliminates one of the primary advantages of using Cas9 over other Cre/LoxP lines. In addition, the need for a dedicated mouse line precludes the flexibility of moving research questions out of mice into other model organisms.

An alternative solution to the size problem involves the use of multiple AAVs, in which one AAV delivers Cas9, whereas another AAV delivers the sgRNA and any other required proteins. This method has been used to drive knockout mutations within the brain and even to multiplex the knockout of three genes simultaneously (Swiech et al., 2015). This method is effective (some labs report 70–80% co-transduction rate) and as such, it may provide a solution for using CRISPRi or CRISPRa *in vivo*, particularly if larger packaging size viruses, such as HSVs, are used.

Finally, homologs of Cas9 exhibit a natural variation in size. Cas9 derived from *Streptococcus aureus* is significantly smaller than the Cas9 from *Streptococcus pyogenes*, the Cas9 discussed thus far. The smaller Cas9 allows the Cas9, sgRNA, and GFP to be packaged together in one AAV (Ran et al., 2015). Together, these solutions and ongoing innovations make CRISPR/Cas9 increasingly accessible for *in vivo* use with various applications in neuroscience.

Although many of the advanced uses of the CRISPR/Cas9 system have not been attempted *in vivo*, the system as a whole is poised to revolutionize gene editing within the brain. With the growth of neuroepigenetics as a field, the use of Cas9 in modulating epigenetic changes in a locus-specific way is ready to take center stage both in neuroscience and in biology in general. With the currently available technologies, neuroscience laboratories can easily start examining single and multi-gene knockouts specifically within the brain. Although the CRISPRi, CRISPRa, and epigenetic modulator methods have not yet been attempted in the nervous system, they are ready for *in vivo* brain validations.

## Disrupting gene expression in the brain without the need for viruses or mouse models

Most modern techniques for modulating gene expression in the brain require the use of an engineered viral vector or mouse model to introduce the gene disruption. Several alternative strategies have been developed to obtain rapid knockdown without the use of viruses, including the use of anti-sense nucleotides, which can be readily purchased for *in vivo* applications, bypassing the need to employ a viral vector or transgenic mice to achieve gene knockdown.

### Use of anti-sense nucleotides for gene disruption in the brain

Anti-sense nucleotides are a widely used method for gene interference. This method relies on relatively short (8–50 bp) oligonucleotides that are complimentary to the sense strand of mRNA. This approach varies from RNAi in that the oligonucleotides are typically composed of DNA rather than RNA, are single stranded, and do not require Dicer processing or incorporation into a RISC (see above). Depending on the targeted location within the transcript, anti-sense mRNA inhibition can be used to induce transcript degradation, inhibit translation, cause aberrant splicing, or interfere with miRNA maturation. The primary goal of these methods is to achieve an inhibition of genes, with the exception of aberrant splicing, which may also be utilized to produce alternative splicing patterns in some transcripts (Passini et al., 2011; Porensky et al., 2012). In most instances, transcript degradation is mediated primarily by recruiting RNase H to degrade DNA–RNA hybrids formed by the binding of anti-sense DNA to its target mRNA (Wheeler et al., 2012), resulting in rapid degradation of the target transcript.

Importantly, the structure of the anti-sense DNA allows these oligonucleotides to be readily taken up by cells in a process called gymnosis, thus eliminating the need for viral vectors, transfection agents, or transgenic mice. However, widespread use of this approach *in vivo* is limited by extensive susceptibility of the oligonucleotides to degradation, marked by the near-ubiquitous presence of nucleases that break their phosphodiester bonds, thereby causing rapid degradation of the anti-sense oligonucleotides (Wickstrom, 1986; Akhtar et al., 1991) and lack of cell specificity. For this reason, research has focused on developing potential modifications that may stabilize oligonucleotides *in vivo* by reducing their susceptibility to degradation, with efforts resulting in two different classes of anti-sense nucleotides. The first major category consists of anti-sense oligonucleotides (ASO) that use various modifications to the existing sugar backbone to prevent degradation, whereas morpholinos represent an additional category that utilizes an entirely different type of backbone to achieve the same goal. Both techniques prevent the molecule from being degraded by traditional nucleases and have progressed to the point of achieving efficient gene inhibition *in vivo* without the need for viral vectors. We discuss each of these categories and their modifications below.

### Using ASOs for direct disruption of gene expression

**Figure 2:**
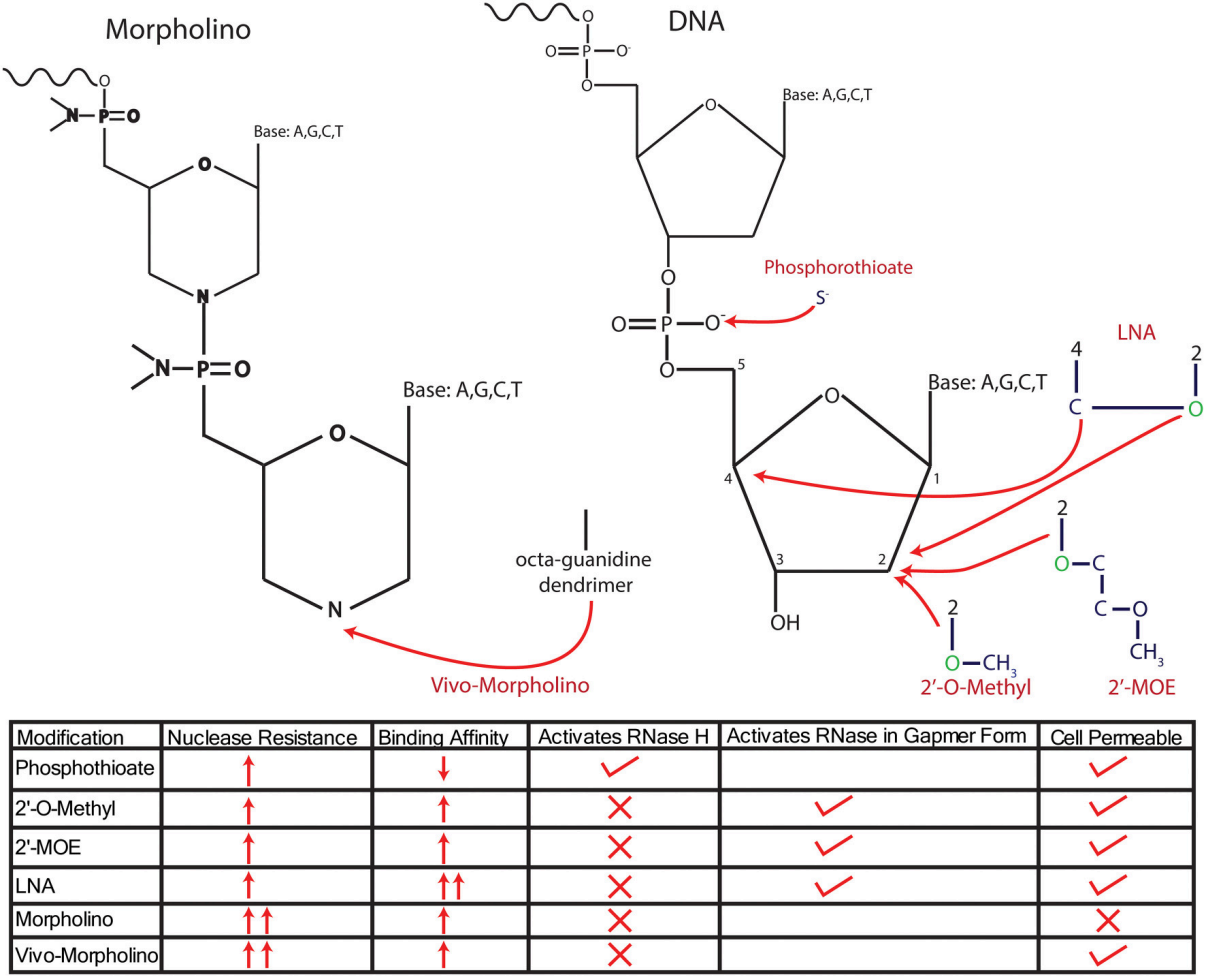
**Modifications of anti-sense nucleotides**. Anti-sense nucleotides are delineated into two major types, anti-sense oligonucleotides (ASOs), which maintain a ribose/deoxyribose backbone, and Morpholinos, which replace the sugar backbone with a morpholine group. ASOs have been modified in several ways to increase their resistance to endogenous nucleases, either by changing the phosphodiester bond or adding molecules onto the sugar backbone itself. Initial modifications altered the phosphodiester bond to a phosphorothioate bond by replacing an O^−^ with an S^−^, resulting in robust nuclease resistance. However, a disadvantage of this modification is the reduced affinity for target mRNA, which has been addressed by combining phosophothioate with modifications to the 2′ C that replace the H with an O-alkyl group, either an O-methyl or O-methyoxy-ethyl (MOE) or an LNA configuration. These modifications further enhance nuclease resistance and increase the affinity of the ASO for mRNA. However, all of these modifications require the addition of oxygen (colored in green) to the 2′ C in the deoxyribose base, thus changing the sugar from deoxyribose into ribose and preventing the recruitment of RNase H to degrade transcripts. Further, modifications designed to recruit RNase H have been achieved by forming “gapmers” that consist of RNA with “gaps” filled by DNA, allowing for successful recruitment of RNase H. Morpholinos reduce nuclease degradation by completely replacing the sugar backbone with a morpholine group. Morpholinos are unable to recruit RNase H, and importantly, they cannot cross the cell membrane without the addition of an octaguanidine dendrimer (*vivo*-Morpholino). The traits of each modification are summed up in the bottom of the panel.

ASOs initiate degradation by binding to target RNA to form DNA–RNA hybrids, which recruit RNase H to degrade the RNA portion of the hybrid (Smith et al., 2006). As mentioned previously, stable knockdown is achieved through several distinct modifications of the sugar backbone or of phosphodiester bonds, with stability and efficacy of the ASO affected by the specific type of modification (see Figure 2). Initial modifications altered the phosphodiester bond to a phosphorothioate bond, thus increasing the half-life of the ASO in serum from 1 to over 9 h [9 h in human serum and up to 19 h in rat CSF (Campbell et al., 1990)]. However, this modification also weakened the bonding affinity of the ASO to its target mRNA, thus necessitating higher doses of ASO and resulting in increased cytotoxicity and off-target effects (Levin, 1999). In this first generation of modified ASOs, the phosphorothioate backbone was still recognized as DNA by RNase H, thus retaining the capacity to cleave the RNA portion of the DNA–RNA hybrid (Wu et al., 2004; Bennett and Swayze, 2010). In addition, this modification also maintains the negative charge of the ASO, allowing it to cross the plasma membrane and be taken up by the cell in its native form.

Successful application of ASOs modified by phosophothioate bonds was reported for both chronic and acute treatment protocols in the brain (Jan et al., 2015), each achieving moderate levels of target knockdown. However, the higher dose and time required for phosphorothioate ASOs to produce efficient knockdown has been associated with neural tissue damage (Engelhard, 1998; Engelhard et al., 1998), thereby limiting their utility. Alternative backbone modifications, such as phosphoramidate bonds, have also been investigated, but their *in vivo* effectiveness in the brain is only a beginning to be investigated (Crooke, 2008). Continuing efforts to develop new modifications, which retain high levels of RNA-binding capacity while achieving high levels of stability, will be a key to more wide-spread use of this technology in neuroscience.

To this end, some success has been reported with other modifications to the ASO sugar ring that increase potency and protect from degradation. One successful approach utilizes a ribose instead of deoxyribose for the sugar moiety, which is then further modified at the 2′ hydroxyl group by adding either a methyl, or the more modern methoxy-ethyl (denoted MOE) group to the 2′ oxygen. These modifications result in improved binding affinity of the ASO, while also protecting it from degradation. However, the conversion of deoxyribose into ribose transforms the oligo into an RNA oligomer, thus precluding the recruitment of RNase H as a mediator of transcript degradation (Baker et al., 1997). That is, RNase H activity is activated in the context of DNA/RNA duplexes, such that conversion of the ASO to RNA produces RNA/RNA duplexes that do not recruit RNaseH (Monia et al., 1993). Although this precludes direct modulation of mRNA degradation, these ASOs can still cause translational inhibition through additional pathways, including selective targeting of the transcript near the translational start site, which results in gene inhibition through steric hindrance of protein translation (Charlier et al., 2005; Osman et al., 2012; Pao et al., 2014). Gene silencing can also be achieved by exon skipping that targets ASOs to specific junctions in the pre-mRNA (Disterer and Khoo, 2012; van Roon-Mom and Aartsma-Rus, 2012), resulting in the production of functionally inert proteins, or steric hindrance of translation (van Roon-Mom and Aartsma-Rus, 2012). An advantage of exon skipping as a knockdown strategy is that it often results in partial knockdown that is relatively amenable to titration (Disterer and Khoo, 2012; van Roon-Mom and Aartsma-Rus, 2012). This strategy is particularly useful for studying many outcome measures in neuroscience that are typically affected by varying levels of gene expression rather than the complete presence or absence of particular proteins.

Alternatively, exon skipping can be utilized to restore function of a previously dysfunctional gene by reinstating a disrupted reading frame, a strategy that is particularly useful for therapeutic intervention in several disease states (Chamberlain and Chamberlain, 2010; van Roon-Mom and Aartsma-Rus, 2012). For example, MOE-modified ASOs have been successfully used in the brain to treat spinal muscular atrophy (SMA), which is caused by null mutations in the *Smn1* gene (Passini et al., 2011). Gene editing efforts are based on the observation that the *Smn1* gene has been duplicated to produce a mostly inactive gene called *Smn2*, which is typically silent due to a splice mutation that prevents the inclusion of exon 7 (Sun et al., 2005). Passini et al. (2011) tested the capacity of MOE-modified ASOs to restore function to the silent *Smn2* gene by targeting the silencing splice mutation in *Smn1* mutant mice. Using intra-ventricular injections of the ASO, the authors found that *Smn2* silencing was reversed through the inclusion of exon 7 into the *Smn2* transcripts, thereby ameliorating SMA pathology (Rigo et al., 2014). Although ASOs are primarily capable of knocking down gene expression, these data demonstrate that ASOs do have a limited capacity to enhance gene activity but only in the context of repairing expression deficits associated with endogenous exon skipping.

### Using locked nucleotides for gene knockdown

A final ASO modification, which has been through several iterations since its introduction, incorporates locked nucleotides (LNA) into the ASO to further increase the binding affinity and knockdown efficiency. LNAs differ from normal nucleotides in that the ribose backbone has been changed to “lock” it into C3′ endo position, thus locking it into a formation that favors normal base pairing (Kurreck et al., 2002). This locking is achieved by connecting the 2′ hydroxyl group, which causes the nucleotide to be recognized as RNA, to the 4′ carbon group to create a stable structure that drastically enhances the binding affinity of the bases and decreases its susceptibility to degradation (Frieden et al., 2003). An important advantage of LNA-based oligos is their retained capacity to cross the blood–brain barrier endogenously, making their delivery into the brain possible. In addition, these oligos may have the most robust defense against degradation and the highest binding affinity among various oligos we have discussed (Wahlestedt et al., 2000; Braasch et al., 2002). However, these modifications still have several major limitations. As discussed in earlier sections, the conversion of oligos to RNA precludes the recruitment of RNase H and requires the use of alternative pathways to achieve gene knockdown. Moreover, LNA-based oligos appear to suffer severe toxicological problems (Kurreck et al., 2002; Swayze et al., 2007; Kakiuchi-Kiyota et al., 2014) and off-target effects (Mook et al., 2010), thus limiting their usefulness *in vivo*.

Much of the current effort is focused on retaining the various 2′ hydroxyl modifications for their capacity to protect oligos from degradation, while also gaining the ability of DNA-based oligos to recruit RNase H when they form a complex with target mRNA. Successful efforts in this search have resulted in a new generation of ASOs that are referred to as “gapmers,” which retain their 2′ hydroxyl groups, but only incorporate them into the first and last five bases of the ASO, thus creating a “gap” of unmodified DNA in the intervening 10 bases (Jepsen and Wengel, 2004; Jepsen et al., 2004; Smith et al., 2006). The hybrid gapmer allows the ASO to benefit from the increased half-life and improved affinity associated with these modifications. However, as the internal 10 bases are DNA, the ASO–mRNA complex is recognized as a DNA–RNA hybrid and degraded by RNase H, thus creating a stable structure that retains RNase H activity. Various iterations of this system exist that are dependent on three main modifications of the 2′ hydroxyl group that increase its stability and binding capacity, as already discussed. The main modifications used in gapmer context include LNAs or the addition of either methyl or MOE groups (Kordasiewicz et al., 2012; Stanek et al., 2013; Mutso et al., 2015).

Gapmers have been successfully used in several *in vivo* applications, including the knockdown of the mutant Huntington's gene (*Htt*) in mouse models of Huntington's disease (Carroll et al., 2011), demonstrating their utility *in vivo*. Specifically, the authors made 2′MOE gapmers with a phosphorothioate backbone and locally injected it into the striatum (Carroll et al., 2011). Two weeks after a single ASO injection, mutant *Htt* protein levels were reduced by 60% and disease progression was delayed (Carroll et al., 2011; Kordasiewicz et al., 2012). Others researchers have found long-lasting decreases in another disease-related gene, *Sod1*, with minimal off-target effects after 28 days of ASO administration directly into the brain (Smith et al., 2006), suggesting their potential utility for achieving stable knockdown *in vivo*.

Overall, LNA-based gapmers have received much attention because of their robust binding affinity. *In vivo*, these gapmers demonstrate impressive results with some ASOs achieving 90% reductions in target transcripts at picomolar concentrations (Dias and Stein, 2002). Unfortunately, the dose required for the majority of ASOs *in vivo* can result in severe toxic side effects (Swayze et al., 2007; Seth et al., 2009), requiring tremendous caution to be used if applying these ASOs in the brain. Better design algorithms, which take LNA–protein interactions into account during the ASO design stage, may be able to minimize these off-target effects, but they still remain the biggest hurdle to wide-spread use of LNA-based gapmers in general and in neuroscience in particular (Koch et al., 2014). Nonetheless, MOE-based gapmers have already demonstrated huge potential for *in vivo* brain research and solidified the gapmer strategy as the best method for future use of ASOs *in vivo*.

## Use of morpholinos to regulate gene expression in the brain

In contrast to ASOs, which take advantage of modifications of the ribose/deoxyribose sugar moiety or phosphodiester bonds as key strategies for improving their stability, morpholinos use a completely synthetic backbone (Summerton and Weller, 1997; Summerton et al., 1997). Morpholinos are oligomers that replace the ribose/deoxyribose sugar ring with a six-member morpholine, which is attached to other morpholines though a non-ionic phosphorodiamidate linkage, which contrasts with the charged phosphodiester bond found in typical ASOs. Each morpholine ring is attached to a base (A,G,C,T), which is free to form normal Watson–Crick base pairs with a target. The exotic backbone of phosphorodiamidate morpholinos oligomer (PMO) prevents it from being recognized and degraded by normal nucleases, allowing the PMO to remain active for long periods of time. As PMOs have such an exotic backbone, they are not recognized as DNA and fail to recruit RNase H upon biding with their target mRNA. However, as with non-gapmer ASOs discussed previously, PMOs can still achieve knockdown by interfering with splicing, translation, and miRNA maturation.

As the phosphorodiamidate bonds in morpholinos do not have a charge, PMOs cannot access the cytoplasm unassisted and require an agent to facilitate their entry. However, the overall uncharged nature of PMOs has also aided their use, in that they show little interaction with cellular proteins and do not appear to elicit an immune response (Moulton, 2013). Despite the general inability to cross cell membranes, PMOs do exhibit unassisted crossing of cell membranes *in vivo* in the mouse model of Duchenne muscular dystrophy, in which cellular membranes are already porous (Ezzat et al., 2015). In this instance, PMOs had a therapeutic effect by causing translational skipping of the *Dmd* transcript, restoring some level of *Dmd* function. However, the inability to cross cell membranes has impeded the use of PMOs outside of this specialized disease, thus limiting their widespread use in modulating genes *in vivo*.

In order to address this limitation, *vivo*-Morpholinos were developed, which consist of a terminal octaguanidinium dendrimer linked to the PMO (Morcos et al., 2008; Reissner et al., 2012). In addition, PPMOs were also developed, which contain cell-penetrating peptides linked to PMO (Stone et al., 2008). Although both modifications are designed to improve PMO uptake into the cytoplasm, *vivo*-Morpholinos have shown utility for *in vivo* brain work (Nizzardo et al., 2014). *vivo*-Morpholinos link the PMO to a large octaguanidinium dendrimer, which results in high cell permeability without reported losses in activity. Many studies have examined the effects of IV or IP injections of the *vivo*-Morpholino and have demonstrated robust inhibition of gene activity (Ferguson et al., 2013; Subbotina et al., 2016). However, uptake of systemically delivered *vivo*-Morpholinos in the brain is much more restricted than in other tissues (Li and Morcos, 2008; Morcos et al., 2008; Parra et al., 2011; Moulton, 2013). Uptake can be dramatically improved by directly injecting *vivo*-Morpholinos into brain regions of interest, thus bypassing the blood–brain barrier and achieving robust suppression of genes for up to 14 days after injection (Oh et al., 2006; Reissner et al., 2012). Importantly, gene suppression was achieved with a relatively low dose of *vivo*-Morpholino (30 pmol), which is significantly below any dose with demonstrated cytotoxic effects (1500 pmol; Reissner et al., 2012).

*vivo*-Morpholinos and gapmer-based ASOs are the first genetic tools that match up with the major advantage of chemical-based antagonists, largely based on their capacity for direct application to a target area without requiring mouse models or viral vectors to achieve knockdown. In addition, ASOs and *vivo*-Morpholinos can be targeted at virtually every transcript and can be designed to accomplish specific tasks, whether that be mRNA degradation, translational inhibition, exon skipping, or inhibiting miRNA processing, a feature that was never available with chemical-based antagonists.

## Conclusion

In recent years, we have seen the development of a wide variety of extremely exciting and highly flexible gene editing approaches that provide entirely new tools for studying gene function in the brain. Here, we highlighted CRISPR/Cas9-based approaches, which offer nearly unlimited flexibility, rapid, and effective targeting of single genes, as well as the ability to target multiple genes simultaneously. Combined with the plethora of available modifications for this system, CRISPR/Cas9 is the most flexible gene editing tool available today. In addition, anti-sense mRNA inhibition is a simple method for obtaining rapid knockdown of a target gene without requiring a viral delivery system, making this approach highly accessible to virtually any lab environment. These two approaches bring powerful new methods into the hands of neuroscientists, providing important tools for answering complex questions regarding gene–behavior relationships in the brain.

## Author contributions

BW, SJ, and IZ developed the concepts and co-wrote the manuscript. AA and CG provided critical research contributions. BW, AA, and CW made the figures. All authors were involved in editing.

## Funding

This work was supported by NSERC grants to SJ and IZ, CIHR to SJ, NSERC CGS-M to CG, and Restracomp Fellowship to BW and CG.

### Conflict of interest statement

The authors declare that the research was conducted in the absence of any commercial or financial relationships that could be construed as a potential conflict of interest.
